# Supplementary material for: Identification of the PTEN-ARID4B-PI3K pathway reveals the dependency on ARID4B by PTEN-deficient prostate cancer
Source: Nat Commun. 2019 Sep 24;10:4332. doi: 10.1038/s41467-019-12184-8 (PMC6760172; doi:10.1038/s41467-019-12184-8)
Supplement: Supplementary file 1 — Supplementary Information [file 41467_2019_12184_MOESM1_ESM.pdf]

Supplementary Information

Identification of the PTEN-ARID4B-PI3K pathway reveals the dependency on ARID4B by PTEN-deficient prostate cancer

Wu et al.

Wu_Fig. S1

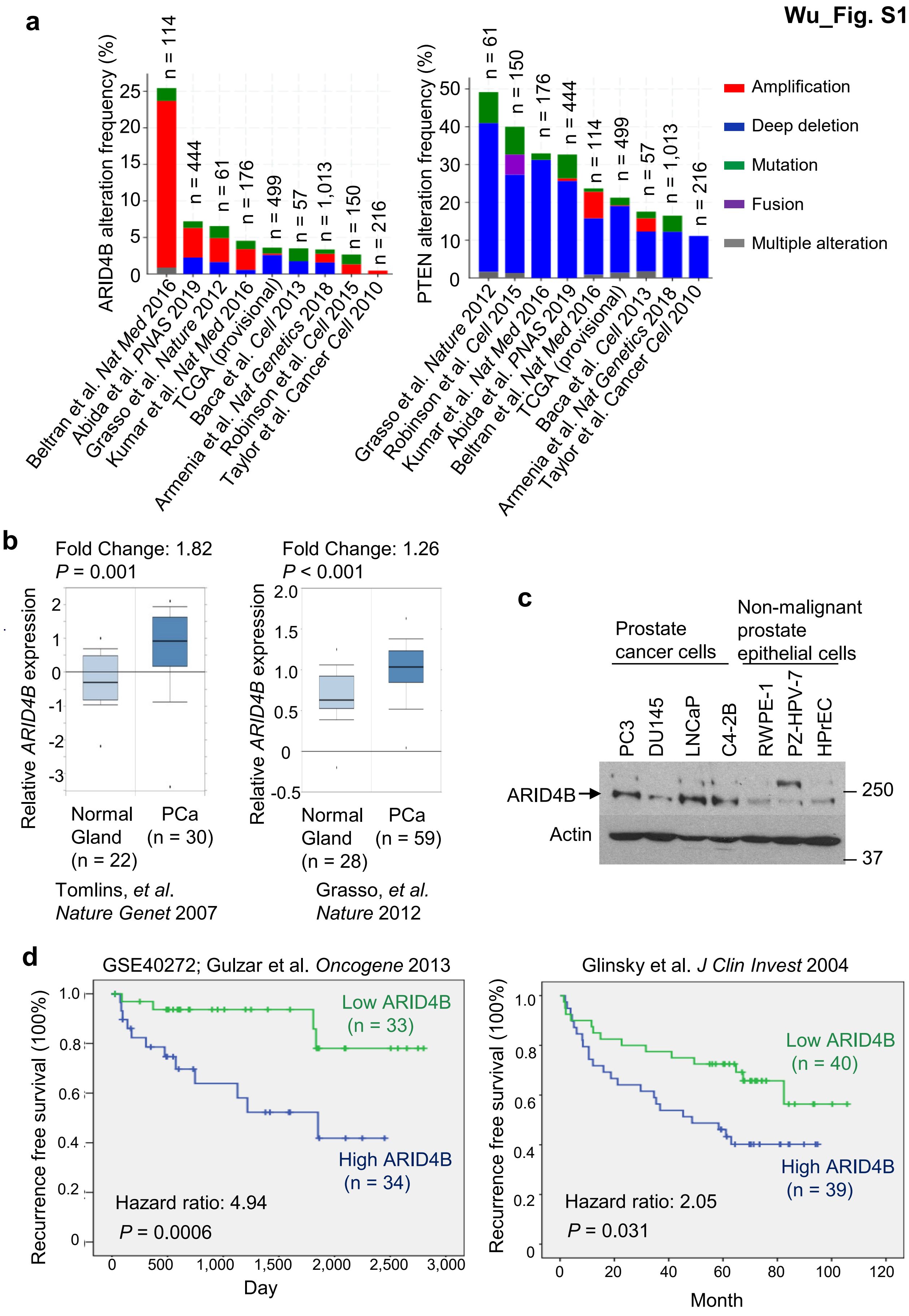

a
ARID4B alteration frequency (%)
n = 114
n = 444
n = 61
n = 176
n = 499
n = 57
n = 1,013
n = 150
n = 216
Beltran et al. Nat Med 2016
Abida et al. PNAS 2019
Grasso et al. Nature 2012
Kumar et al. Nat Med 2016
TCGA (provisional)
Baca et al. Cell 2013
Armenia et al. Nat Genetics 2018
Robinson et al. Cell 2015
Taylor et al. Cancer Cell 2010
PTEN alteration frequency (%)
Amplification
Deep deletion
Mutation
Fusion
Multiple alteration
b
Fold Change: 1.82
P = 0.001
Relative ARID4B expression
Normal Gland (n = 22)
PCa (n = 30)
Tomlins, et al. Nature Genet 2007
Fold Change: 1.26
P < 0.001
Normal Gland (n = 28)
PCa (n = 59)
Grasso, et al. Nature 2012
c
Prostate cancer cells
Non-malignant prostate epithelial cells
PC3
DU145
LNCaP
C4-2B
RWPE-1
PZ-HPV-7
HPrEC
ARID4B
Actin
250
37
d
GSE40272; Gulzar et al. Oncogene 2013
Low ARID4B (n = 33)
High ARID4B (n = 34)
Hazard ratio: 4.94
P = 0.0006
Recurrence free survival (100%)
Day
Glinsky et al. J Clin Invest 2004
Low ARID4B (n = 40)
High ARID4B (n = 39)
Hazard ratio: 2.05
P = 0.031
Month

Supplementary Fig. 1 Expression of *ARID4B* is increased in human prostate cancer and negatively correlated with recurrence free survival. **a,** Frequency of genomic alterations on *ARID4B* (left) and *PTEN* (right) in the nine prostate cancer genomic datasets. **b,** Boxed plot comparing the *ARID4B* expression levels between human normal prostate and prostate carcinoma (PCa) from two datasets using Oncomine analysis. Statistical analysis: *t*-test. **c,** The relative expression levels of ARID4B in human prostate cancer cell lines (PC3, DU145, LNCaP, and C4-2B) and non-malignant prostate epithelial cell lines (RWPE-1, PZ-HPV-7, and HPrEC) was analyzed by western blot. **d,** Kaplan-Meier survival analysis shows the correlation between recurrence free survival and the *ARID4B* expression index in prostate cancer patients from two datasets. Statistical analysis: log-rank test.

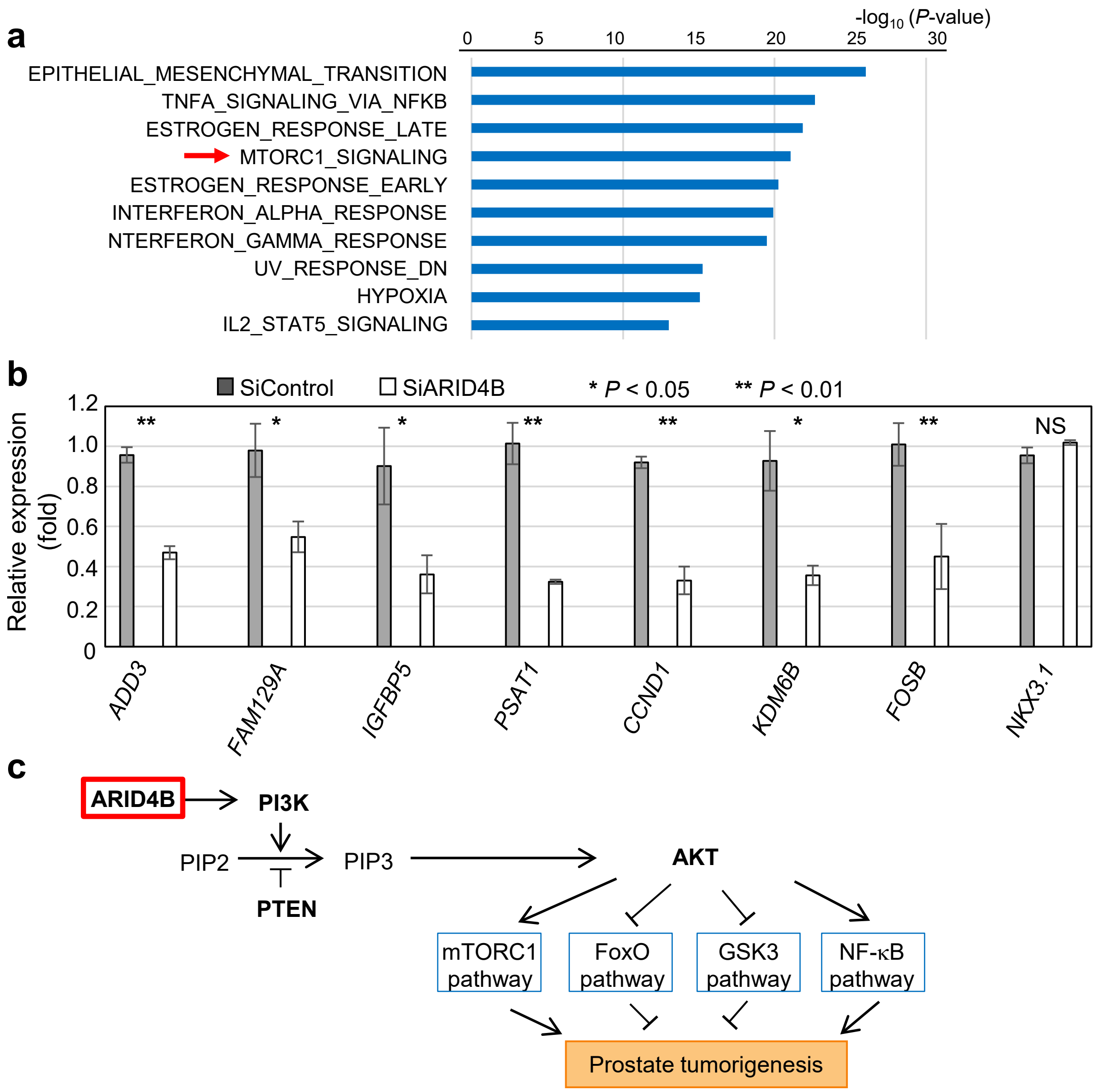

Supplementary Fig. 2 ARID4B regulates the PTEN/PI3K/AKT pathway. **a,** Gene set enrichment analysis (GSEA) shows top ten hallmark pathways of ARID4B-regulated genes identified by RNA-Seq that was performed to analyze gene expression profiles in PC3 cells transfected with SiControl or SiARID4B. Graph displays category scores as $-\log_{10}$(P value). Statistical analysis: Wald Chi-Squared test. **b,** qRT-PCR analyses confirmed the reduced mRNA levels of *ADD3*, *FAM129A*, *IGFBP5*, *PSAT1*, *CCND1*, *KDM6B*, and *FOSB* in the Si*ARID4B* PC3 cells. The mRNA level of *NKX3.1* in Si*ARID4B* PC3 cells is comparable with that in SiControl PC3 cells. RNA from three samples of PC3 cells transfected with SiControl or Si*ARID4B* were analyzed. The level of gene expression from one sample of PC3 cells transfected with SiControl was set as 1. Data are means ± SEM. *, $P < 0.05$; **, $P < 0.01$; Statistical analysis: *t*-test. **c,** A schematic representation of the role of ARID4B in the PTEN/PI3K/AKT signaling network and prostate tumorigenesis.

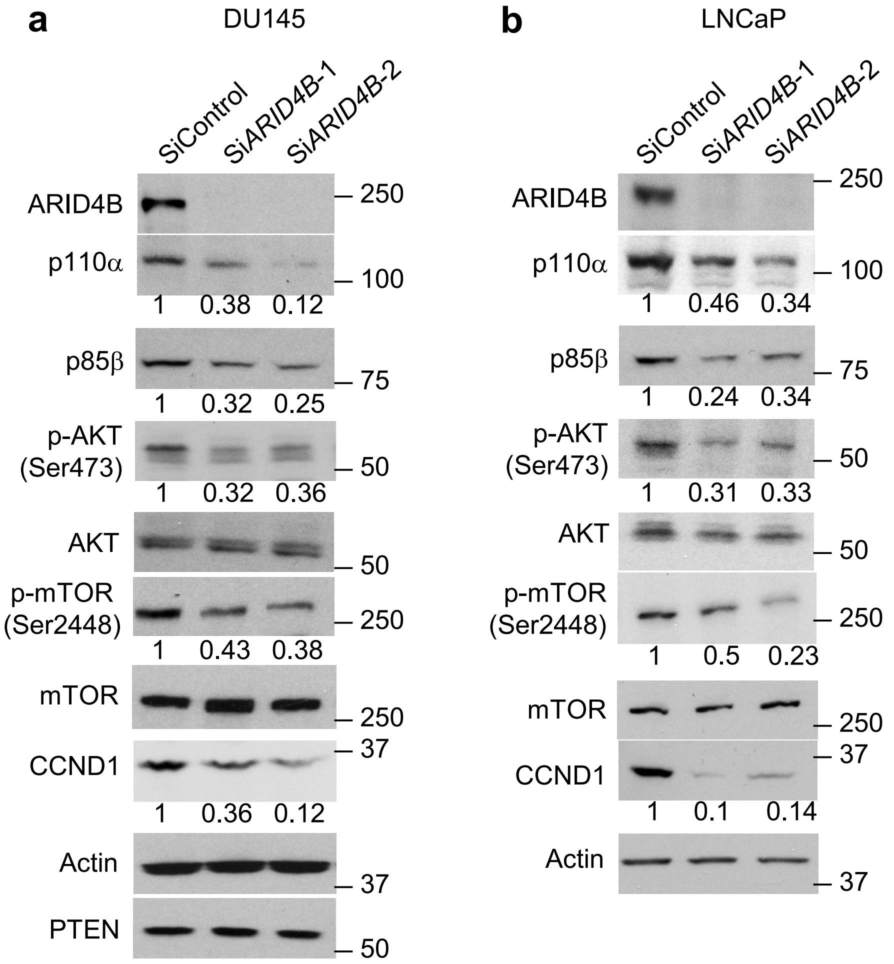

Supplementary Fig. 3 Ablation of ARID4B inhibits the PI3K-AKT pathway in prostate cancer cell lines. **a, b,** Expression or phosphorylation of core regulators and downstream effectors of the PI3K-AKT pathway in DU145 (**a**) and LNCaP (**b**) cells transfected with SiControl, Si*ARID4B*-1, or Si*ARID4B*-2 were analyzed by western blot. The intensity of images was measured by Image J software. The intensity level of image from the SiControl sample was set as 1.

Wu_Fig. S4

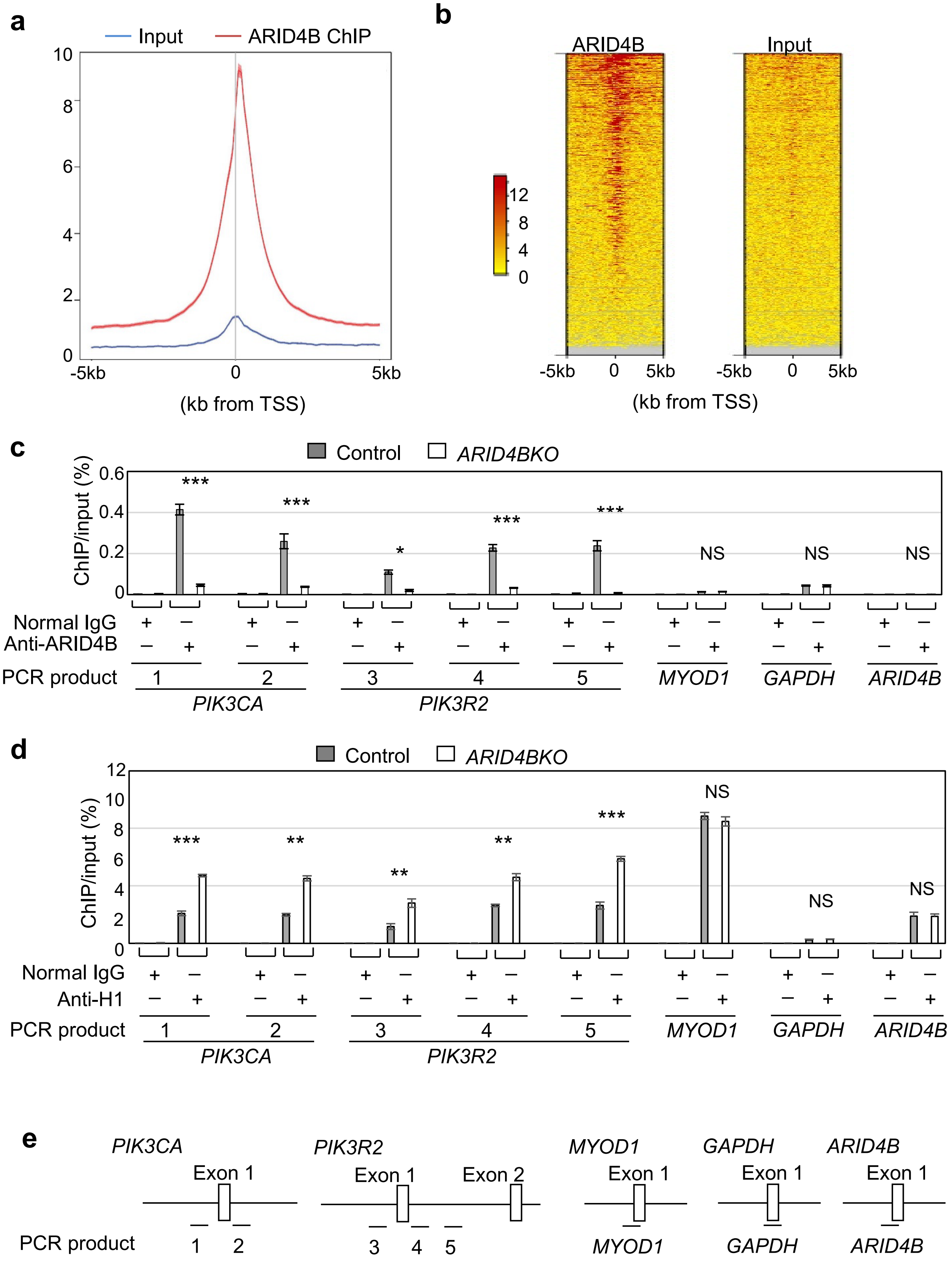
a
Input
ARID4B ChIP
10
8
6
4
2
0
-5kb
0
5kb
(kb from TSS)
b
ARID4B
Input
12
8
4
0
-5kb
0
5kb
-5kb
0
5kb
(kb from TSS)
c
Control
ARID4BKO
ChIP/input (%)
0.6
0.4
0.2
0
***
***
*
***
***
NS
NS
NS
Normal IgG
Anti-ARID4B
PCR product
1
2
3
4
5
MYOD1
GAPDH
ARID4B
PIK3CA
PIK3R2
d
Control
ARID4BKO
ChIP/input (%)
12
10
8
6
4
2
0
***
**
**
**
***
NS
NS
NS
Normal IgG
Anti-H1
PCR product
1
2
3
4
5
MYOD1
GAPDH
ARID4B
PIK3CA
PIK3R2
e
PIK3CA
PIK3R2
MYOD1
GAPDH
ARID4B
Exon 1
Exon 1
Exon 2
Exon 1
Exon 1
Exon 1
PCR product
1
2
3
4
5
MYOD1
GAPDH
ARID4B

Supplementary Fig. 4 Genome-wide analysis of ARID4B binding. **a,** The promoter average plot from ChIP-Seq analysis using anti-ARID4B antibody showed that the promoter enrichment peak of ARID4B ChIP is markedly stronger compared to the peak of the input control. **b,** Heat maps of ChIP-Seq data show enrichment of ARID4B binding within +/- 5Kb of transcriptional start sites (TSSs) of genes compared to input. **c, d,** The recruitments of ARID4B (**c**) and histone H1 (**d**) to the promoters of *PIK3CA*, *PIK3R2*, *MYOD1*, *GAPDH*, and *ARID4B* in control and *ARID4B*KO PC3 cells were analyzed by ChIP-qPCR analyses using anti-ARID4B (**c**) or anti-H1 (**d**) antibodies. In each case, normal rabbit IgG was used for comparison. Data are means ± SEM from three experiments performed in triplicate. *, $P < 0.05$; **, $P < 0.01$; ***, $P < 0.001$; NS, no significant differences; Statistical analysis: *t*-test. **e,** The PCR products on the promoter regions of *PIK3CA*, *PIK3R2*, *MYOD1*, *GAPDH*, and *ARID4B* detected by specific primer sets used in ChIP-qPCR analysis are as indicated.

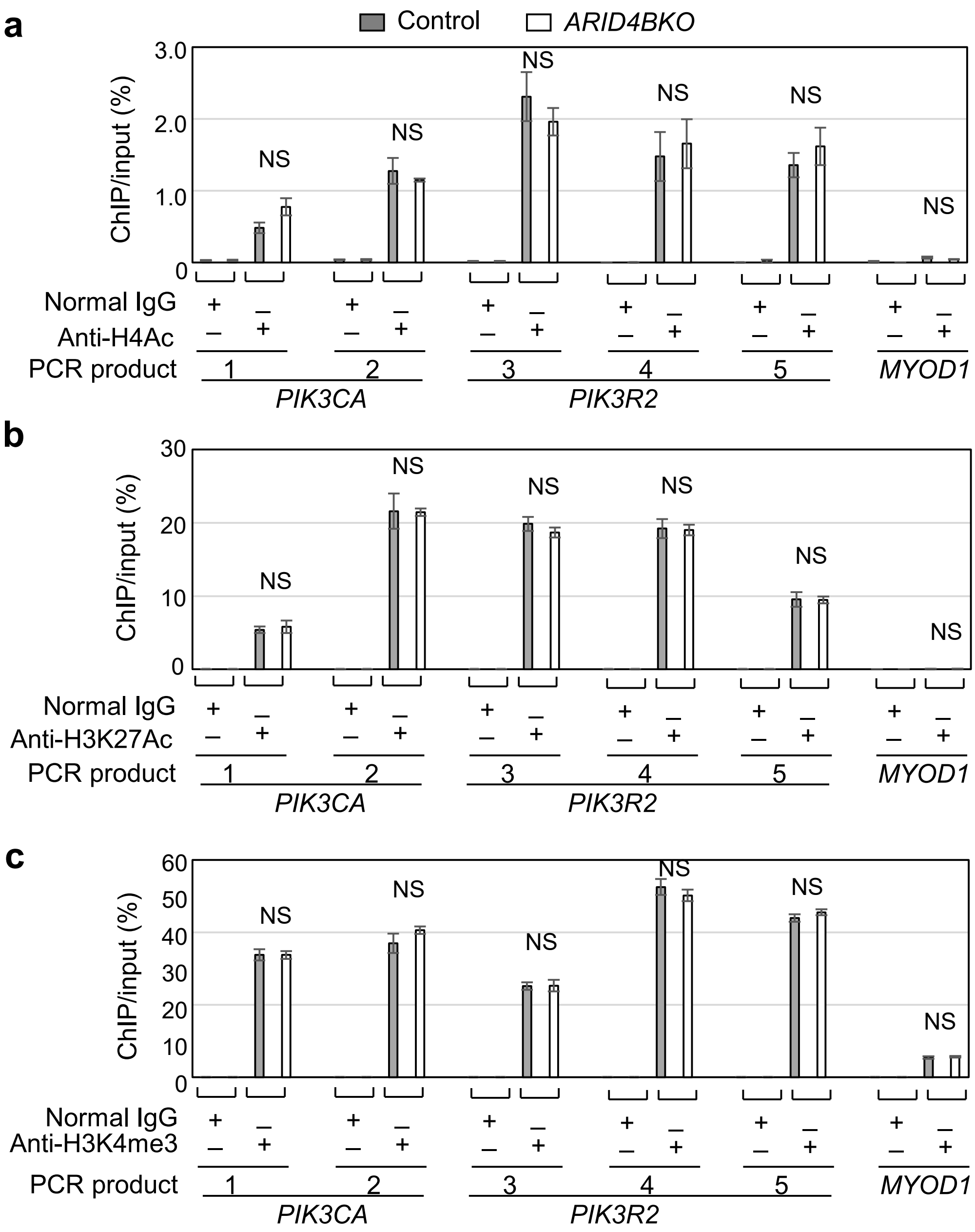

Supplementary Fig. 5 Chromatin modifications on the *PIK3CA* and *PIK3R2* promoters in the control and *ARID4B*KO PC3 cells. **a-c,** ChIP-qPCR analyses showed no difference in histone H4 acetylation (H4Ac) (**a**), H3 lysine 27 acetylation (H3K27Ac) (**b**), and H3 lysine 4 trimethylation (H3K4me3) (**c**) on the promoters of *PIK3CA*, *PIK3R2*, and *MYOD1* between control and *ARID4B*KO PC3 cells. The ChIP assays were performed using anti-H4Ac, anti-H3K27Ac, or anti-H3K4me3 antibodies. qPCR analysis used the primer sets for the PCR products in the promoter regions of *PIK3CA* (PCR products 1 and 2), *PIK3R2* (PCR products 3 - 5), and *MYOD1*. See Supplementary Fig. 4e for more information on the region of each qPCR product. In each case, normal rabbit IgG was used for comparison. Data are means ± SEM from three experiments performed in triplicate. NS, no significant differences; Statistical analysis: *t*-test.

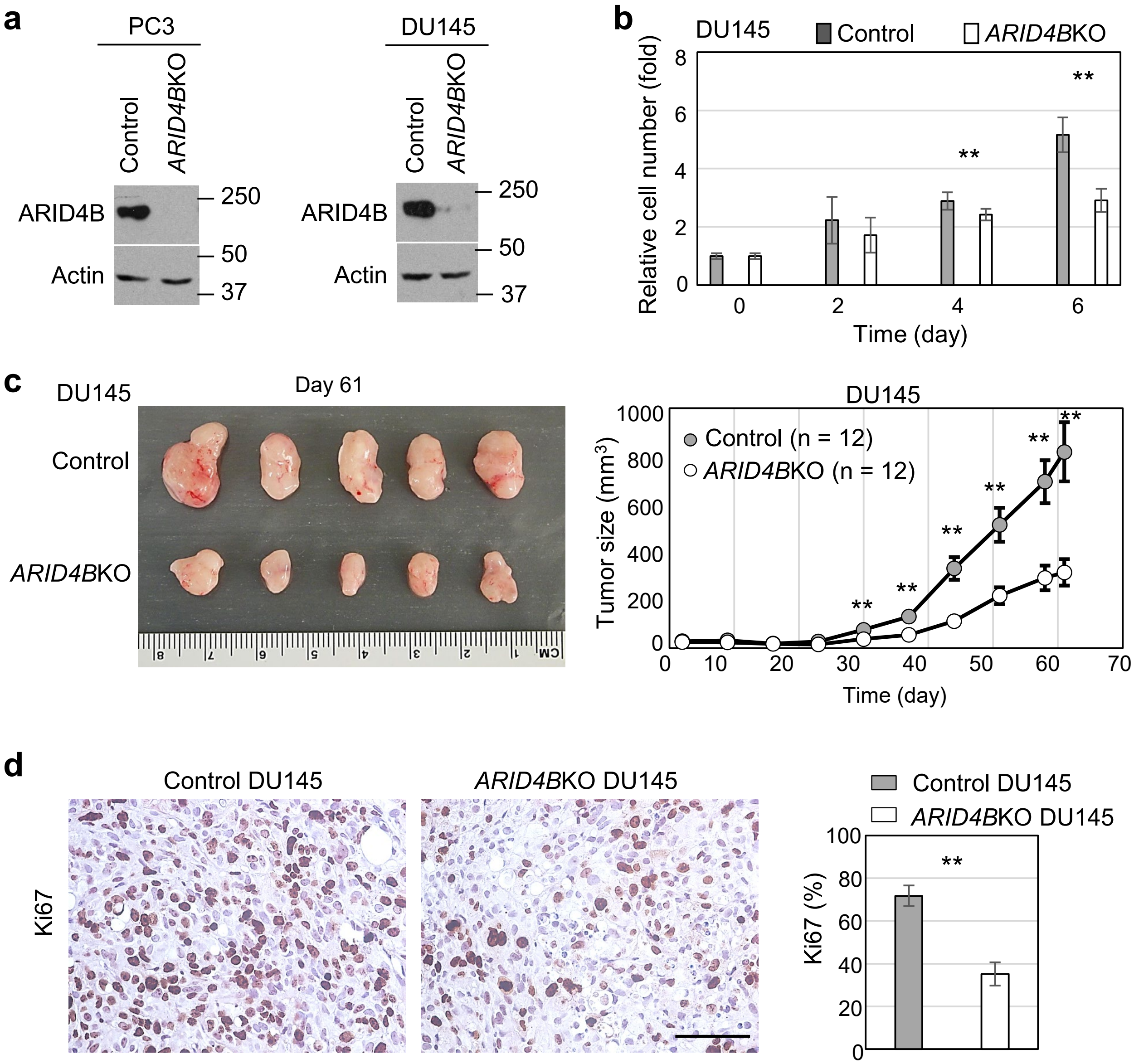

Supplementary Fig. 6 Ablation of ARID4B in PTEN-intact prostate cancer DU145 cells moderately suppressed tumor growth. **a,** CRISPR-mediated knockout of *ARID4B* in PC3 and DU145 cells was confirmed by western blot analysis (compare control and *ARID4B*KO cells). **b,** Proliferation of control and *ARID4B*KO DU145 cells was measured by MTT assays. **c,** Representative images of excised tumors from xenograft experiments (left) and measurement of tumor growth (right) in mice injected with control or *ARID4B*KO DU145 cells. **d,** Representative images (left) and quantification (right) of immunohistochemical Ki67 staining on excised tumors from mice injected with control or *ARID4B*KO DU145 cells (n = 3 for each group). Scale bar, 50 µm. Data are means ± SEM from three experiments performed in triplicate (**b**, **d**). **, $P < 0.01$ (**b-d**); Statistical analysis: *t*-test.

Wu_Fig. S7

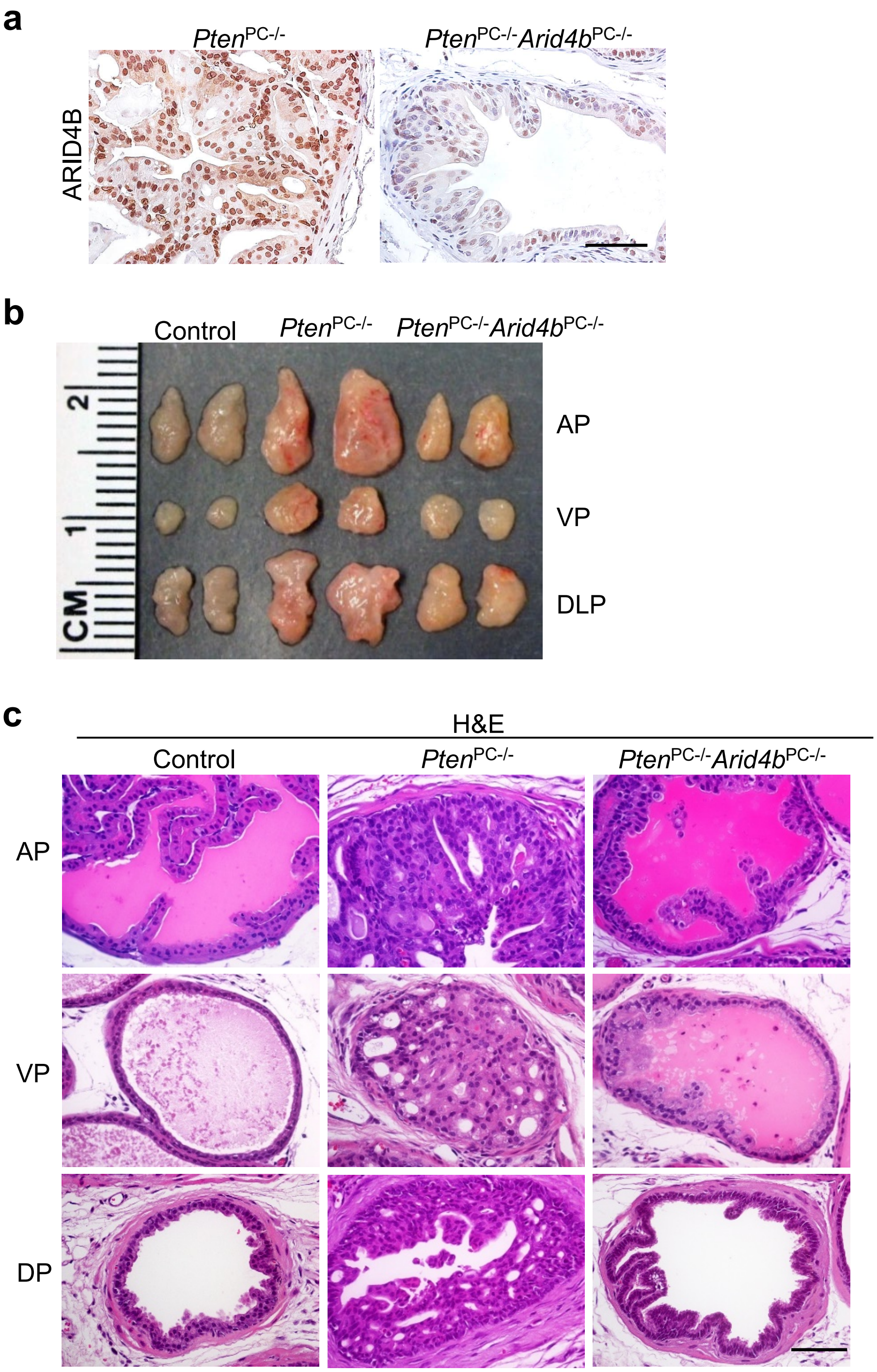

Supplementary Fig. 7 Ablation of ARID4B suppressed progression of prostate cancer elicited by PTEN deficiency in mice. **a,** Expression of ARID4B in anterior prostate of the *Pten*$^{PC-/-}$ and *Pten*$^{PC-/-}$*Arid4b*$^{PC-/-}$ mice at 5 months of age was analyzed by immunohistochemical staining**. b, c,** Representative images of the prostate lobes (**b**) and haematoxylin and eosin (H&E) stained sections of prostates (**c**) from the control, *Pten*$^{PC-/-}$, and *Pten*$^{PC-/-}$*Arid4b*$^{PC-/-}$ mice at 5 months of age. AP, anterior prostate; VP, ventral prostate; DP, dorsal prostate. Scale bar, 50 μm (**a**, **c**).

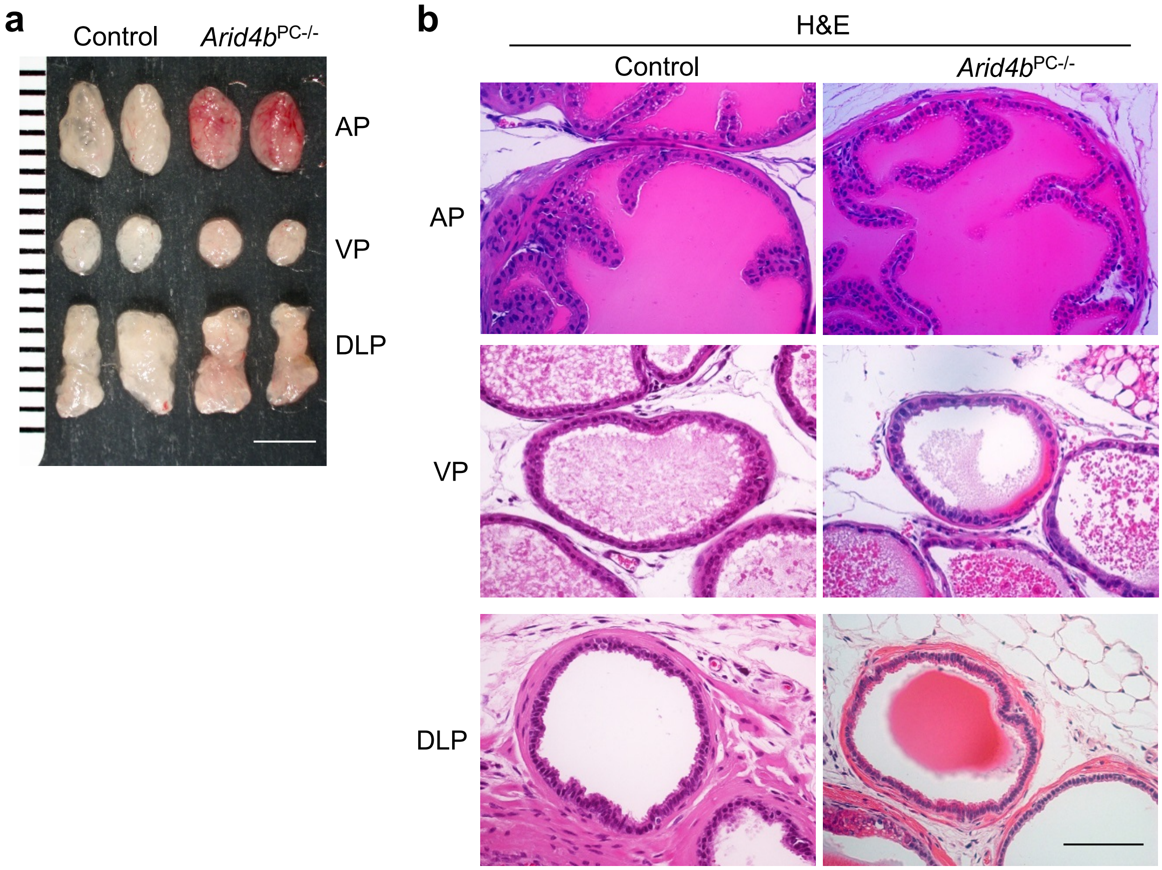

Supplementary Fig. 8 Normal prostate size and histology in the *Arid4b*$^{PC-/-}$ mice. **a, b,** Representative images of the prostate lobes (**a**) and haematoxylin and eosin (H&E) stained sections of prostates (**b**) from the control and *Arid4b*$^{PC-/-}$ mice at 5 months of age. Scale bars, 3 mm (**a**) and 50 μm (**b**). AP, anterior prostate; VP, ventral prostate; DLP, dorsal-lateral prostate.

Wu_Fig. S9

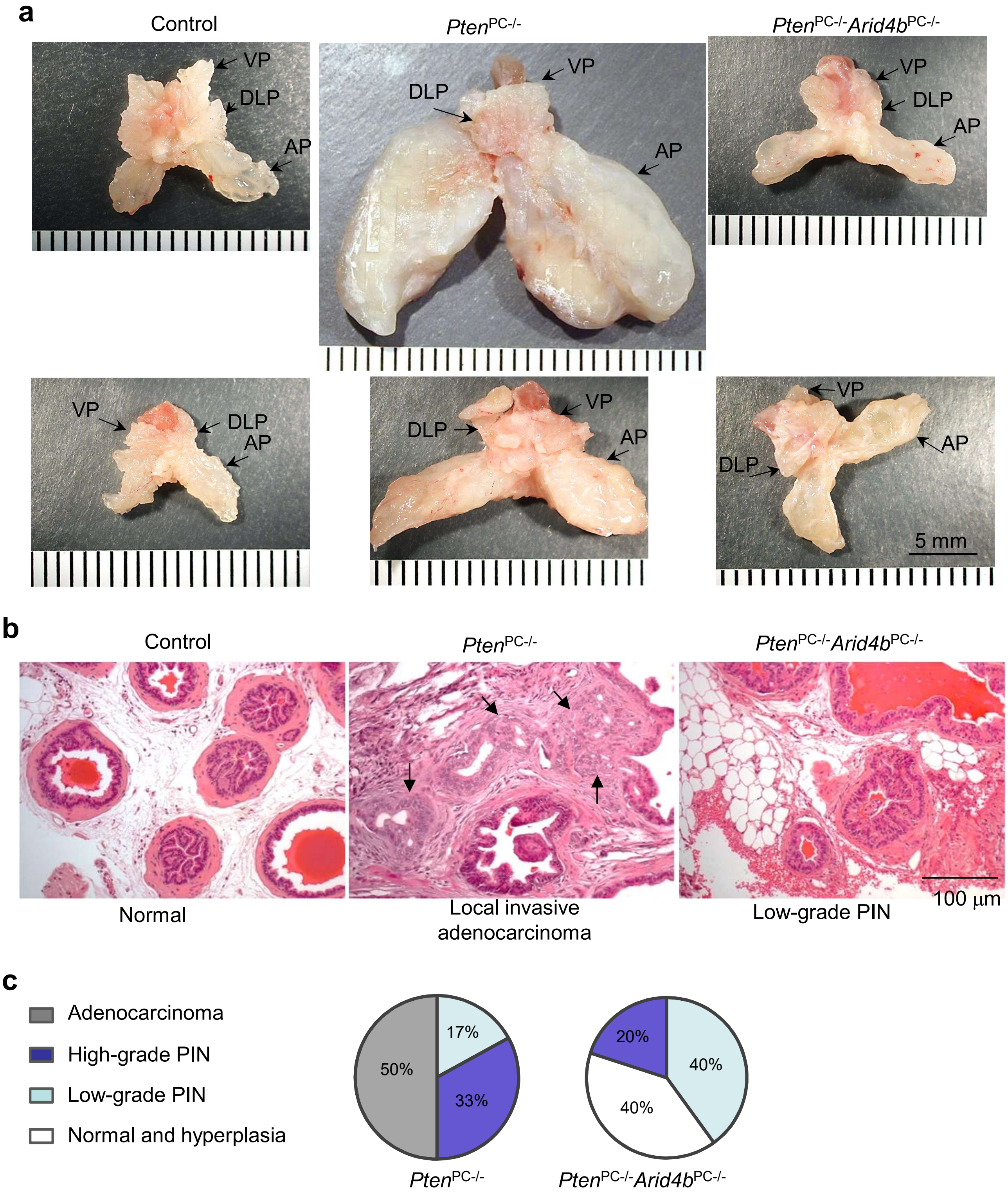
a
Control
Pten^PC-/-
Pten^PC-/- Arid4b^PC-/-
VP
DLP
AP
5 mm
b
Control
Pten^PC-/-
Pten^PC-/- Arid4b^PC-/-
Normal
Local invasive adenocarcinoma
Low-grade PIN
100 µm
c
Adenocarcinoma
High-grade PIN
Low-grade PIN
Normal and hyperplasia
50%
17%
33%
20%
40%
40%
Pten^PC-/-
Pten^PC-/- Arid4b^PC-/-

Supplementary Fig. 9 The *Pten*$^{PC-/-}$*Arid4b*$^{PC-/-}$ mice did not develop prostate cancer at 9 month of age. **a,** Gross anatomy of prostates from the control, *Pten*$^{PC-/-}$, and *Pten*$^{PC-/-}$*Arid4b*$^{PC-/-}$ mice at 9 months of age. While the *Pten*$^{PC-/-}$ mice exhibited enlarged prostates, the *Pten*$^{PC-/-}$*Arid4b*$^{PC-/-}$ mice showed comparable prostate size with the control mice. AP, anterior prostate; VP, ventral prostate; DLP, dorsal-lateral prostate. **b,** Haematoxylin and eosin staining of the dorsal prostates from the control, *Pten*$^{PC-/-}$, and *Pten*$^{PC-/-}$*Arid4b*$^{PC-/-}$ mice at 9 months of age. Arrows point to local invasive adenocarcinoma in the *Pten*$^{PC-/-}$ prostate. PIN, prostatic intraepithelial neoplasia. **c,** Pie graphs are used to summarize prostate tumor progression in the *Pten*$^{PC-/-}$ (n = 6) and *Pten*$^{PC-/-}$*Arid4b*$^{PC-/-}$ mice (n = 5) at 9 months of age.

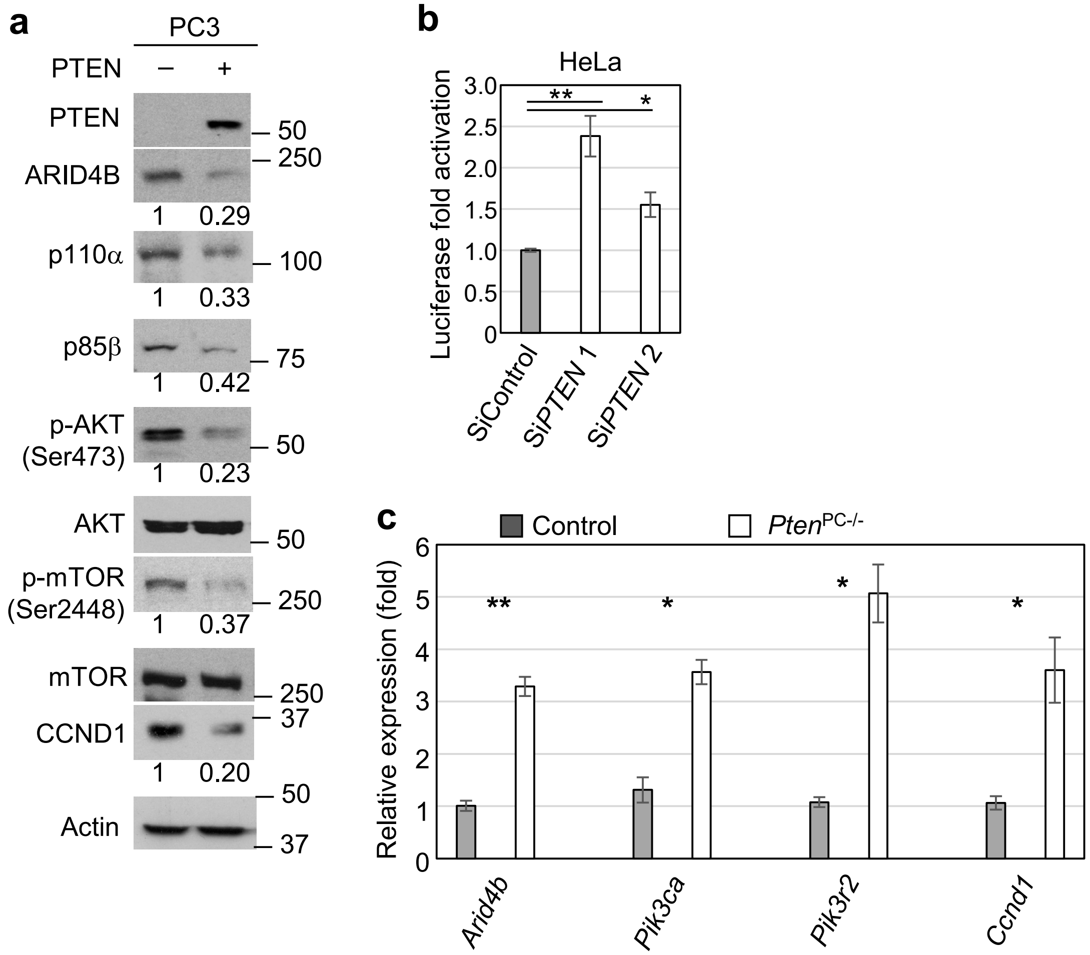

Supplementary Fig. 10 PTEN negatively regulates expression of ARID4B. **a,** Expression or phosphorylation of core regulators and downstream effectors of the PTEN-PI3K-AKT pathway in PC3 cells with or without re-expression of PTEN were determined by western blot analysis. The intensity of images was measured by Image J software. The intensity level of image from the PC3 cells without re-expression of PTEN was set as 1. **b,** Knockdown of *PTEN* by two different short interfering RNA Si*PTEN* 1 and Si*PTEN* 2 increased activity of the *ARID4B* promoter. Luciferase reporter assay using the *ARID4B* promoter-driven luciferase reporter was performed in PTEN-intact HeLa cells. Data are means ± SEM from three experiments performed in triplicate. **c,** The mRNA levels of *Arid4b*, *Pik3ca*, *Pik3r2*, and *Ccnd1*, in the control and *Pten*$^{PC-/-}$ mice at 7 months of age were determined by qRT-PCR analyses. Results are from three experiments performed in triplicate. The level of gene expression from one sample of the control mice was set as 1. Data are means ± SEM. *, $P < 0.05$; **, $P < 0.01$; Statistical analysis: *t*-test.

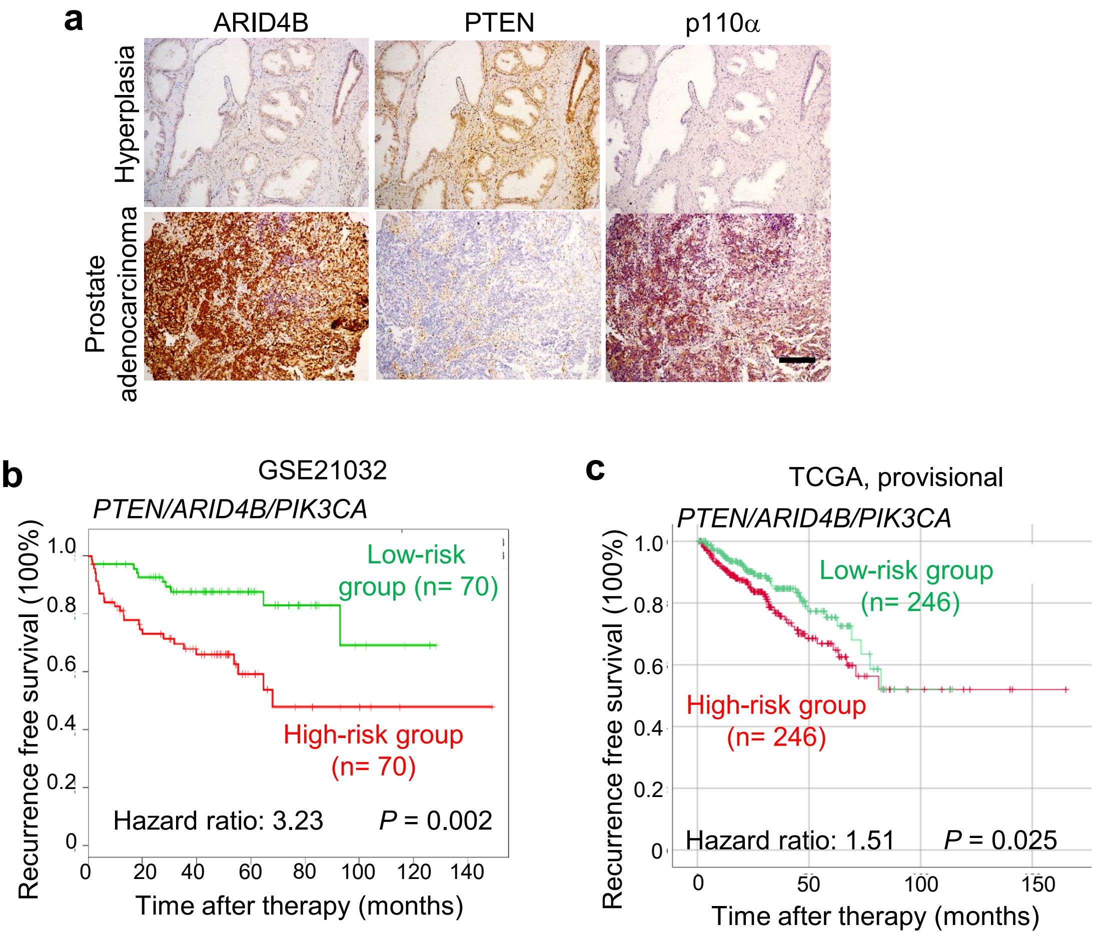

Supplementary Fig. 11 The *ARID4B/PIK3CA/PTEN* three-gene signature provides increased predictive power for prostate cancer recurrence. **a,** Representative immunohistochemical staining of ARID4B, PTEN, and p110α from one prostate hyperplasia patient (top) and one prostate adenocarcinoma patient (bottom) in TMA. Scale bar, 200μm. **b, c,** Kaplan-Meier plot of prostate cancer recurrence free survial in patients from the GSE21032 (b) and TCGA provisional (c) datasets stratified by the *PTEN/ARID4B/PIK3CA* three-gene combinatorial signature. Statistical analysis: log-rank test.

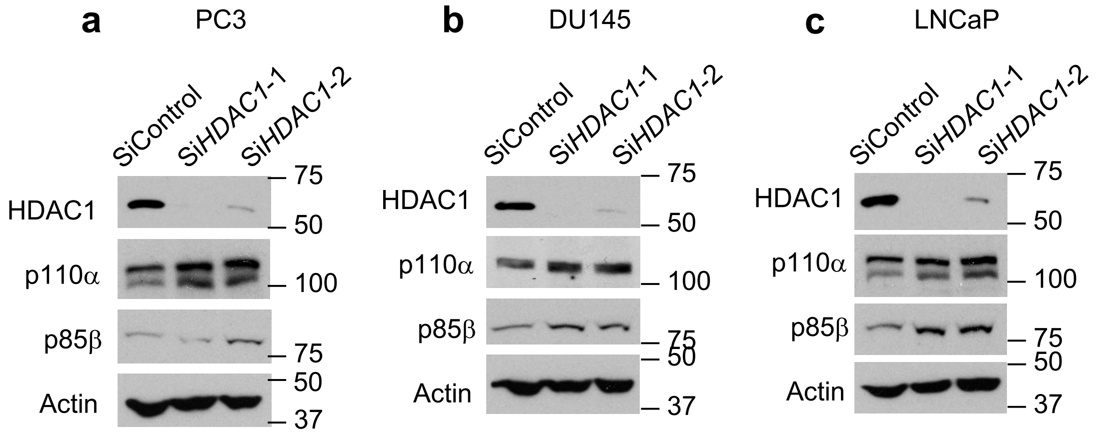

Supplementary Fig. 12 Depletion of *HDAC1* increased expression of p110α and p85β in prostate cancer cell lines. **a-c,** Expression of HDAC1, p110α, and p85β in PC3 (**a**), DU145 (**b**), and LNCaP (**c**) cells transfected with SiControl, Si*HDAC1*-1, or Si*HDAC1*-2 was analyzed by western blot.

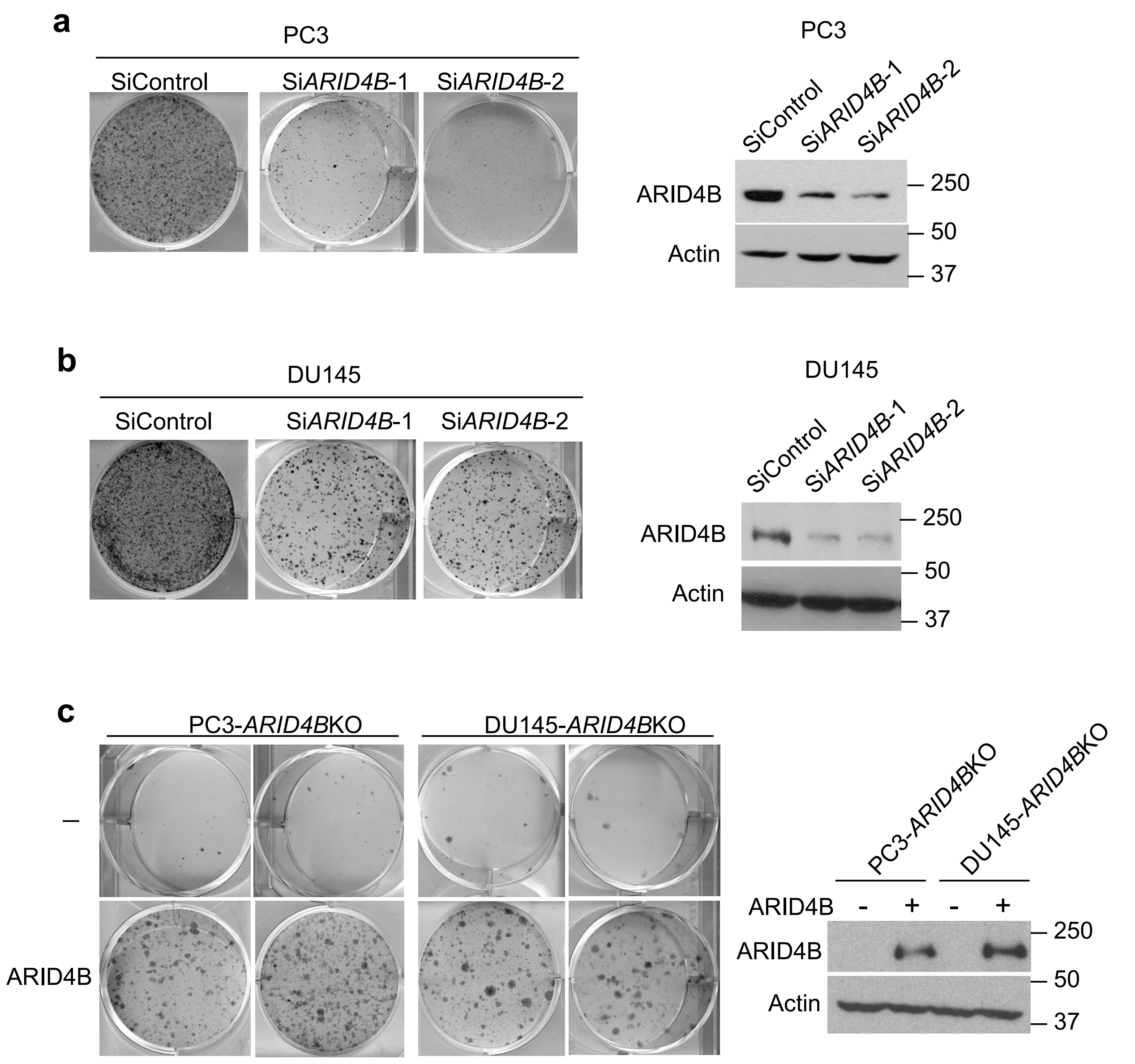

Supplementary Fig. 13 Ablation of ARID4B suppressed the activity of colony formation in prostate cancer cells PC3 and DU145. **a,b,** Colony formation assays were performed in PC3 (**a**, left) and DU145 (**b**, left) cells transfected with SiControl, Si*ARID4B*-1, or Si*ARID4B*-2. Knockdown of *ARID4B* in PC3 (**a**) and DU145 (**b**) cells by Si*ARID4B*-1 or Si*ARID4B*-2 was confirmed by western blot analyses (right). **c,** Re-expression of *ARID4B* in PC3 and DU145 cells with knockout of *ARID4B* (PC3-*ARID4B*KO and DU145-*ARID4B*KO, respectively) promotes colony formation (left). Re-expression of *ARID4B* in PC3-*ARID4B*KO and DU145-*ARID4B*KO cells was confirmed by western blot analyses (right).

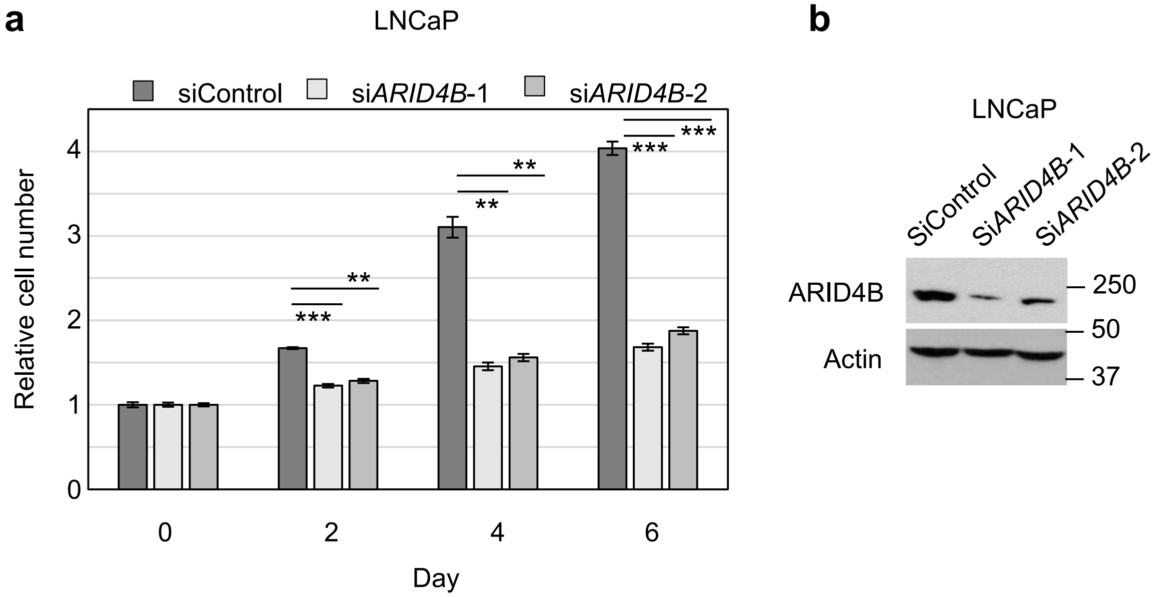

Supplementary Fig. 14 Knockdown of *ARID4B* in PTEN-deficient prostate cancer LNCaP cells suppressed cell growth. **a,** Proliferation of LNCaP cells transfected with SiControl, Si*ARID4B*-1, or Si*ARID4B*-2 was measured by MTT assays at the days indicated. Data are means ± SEM from three experiments performed in triplicate. ***, $P < 0.001$; **, $P < 0.01$; *, $P < 0.05$; Statistical analysis: *t*-test. **b,** Knockdown of *ARID4B* in LNCaP cells by Si*ARID4B*-1 or Si*ARID4B*-2 was confirmed by western blot analysis.

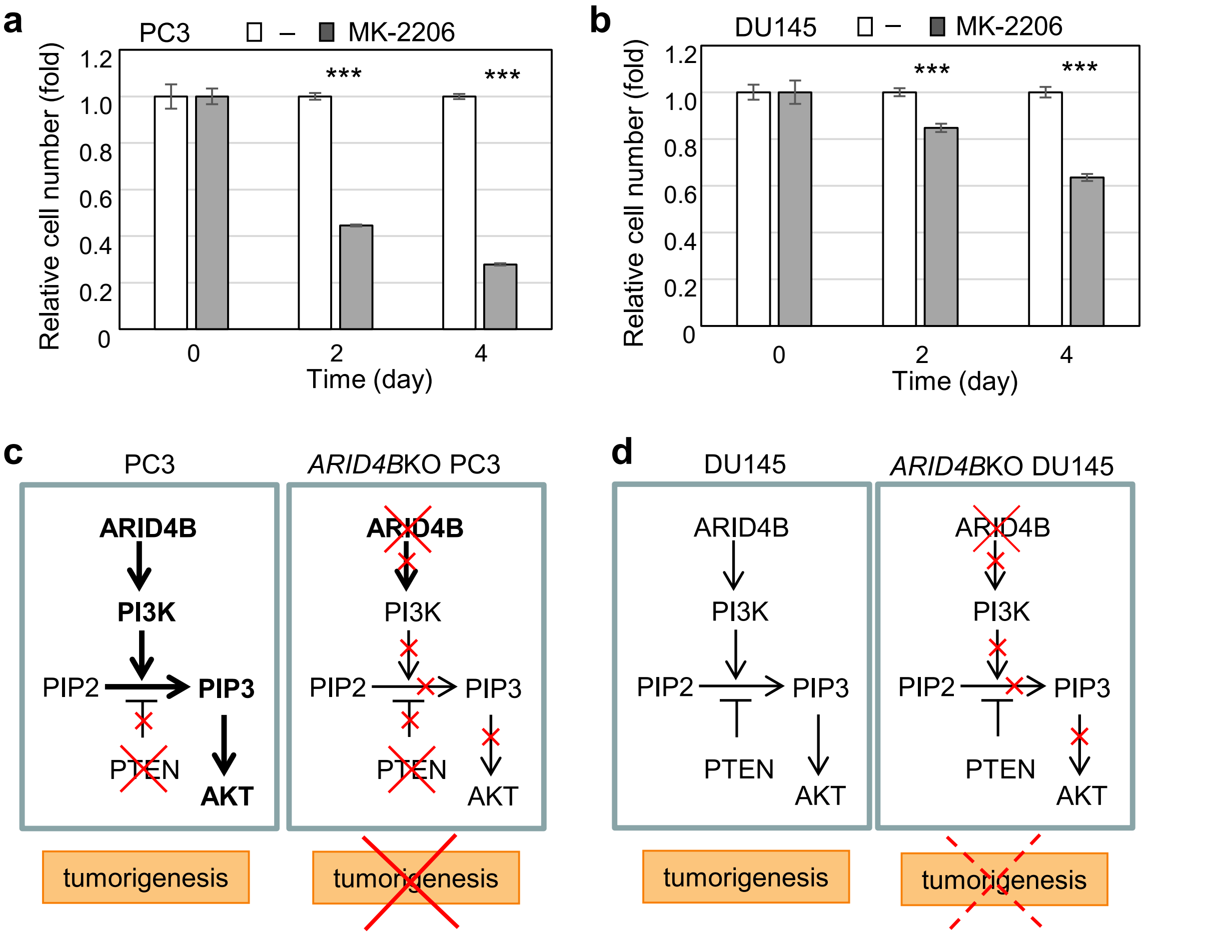

Supplementary Fig. 15 PTEN-intact DU145 cells is less dependent on activation of the PI3K-AKT pathway than PTEN-deficient PC3 cells. **a, b,** Proliferation of PC3 (**a**) and DU145 (**b**) cells between untreated or treaed with an AKT inhibitor MK-2206 (10 μM) were compared. Cell number was measured by MTT assay. The number of one untreated sample was set as 1. Data are means ± SEM from three experiments performed in triplicate. ***, $P < 0.001$; Statistical analysis: *t*-test. **c,** In PTEN-deficient PC3 cells, ablation of PTEN results in activation of the PI3K/AKT pathway, leading to tumorigenesis (left). In the *ARID4B*KO PC3 cells, knockout of *ARID4B* compromises activation of the PI3K/AKT pathway, thus efficiently inhibiting tumorigenesis elicited by PTEN deficiency (right). **d,** In the PTEN-intact DU145 cells, PTEN dephosphorylates PIP3 into PIP2, which opposes the PI3K/AKT signaling pathway (left), suggesting tumorigenesis of DU145 cells is less dependent on the PI3K-AKT signaling than PC3 cells. Therefore, knockout of *ARID4B* that inactivates the PI3K/AKT pathway only moderately suppressed tumorigenesis of DU145 cells (right).

Wu_Fig. S16

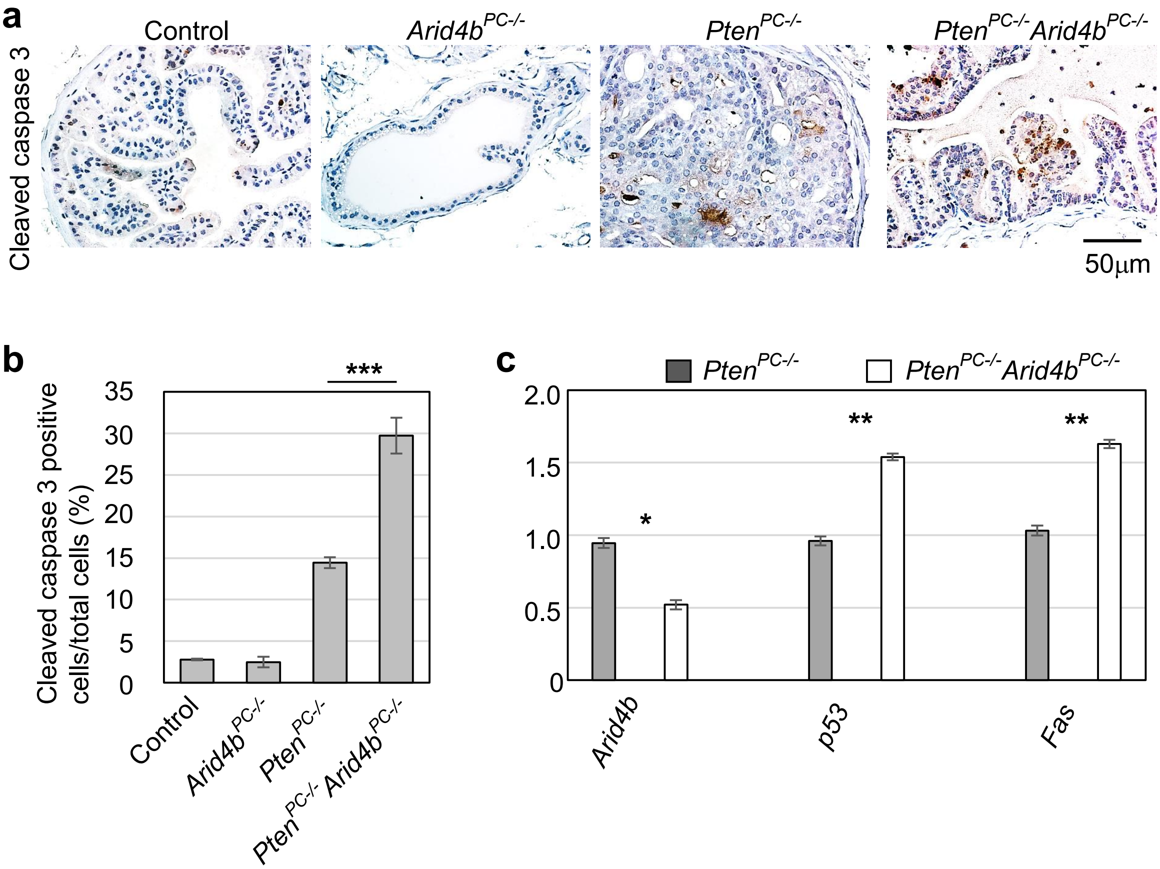

Supplementary Fig. 16 Increased apoptosis in the *Pten*$^{PC-/-}$*Arid4b*$^{PC-/-}$ prostates. **a,** Detection of apoptosis by immunohistochemical staining using the anti-cleaved Caspase 3 antibody in prostates from the control, *Arid4b*$^{PC-/-}$, *Pten*$^{PC-/-}$, and *Pten*$^{PC-/-}$*Arid4b*$^{PC-/-}$ mice at 5 months of age. **b,** Quantification of the cleaved caspase 3 positive cells in prostates from the control, *Arid4b*$^{PC-/-}$, *Pten*$^{PC-/-}$, and *Pten*$^{PC-/-}$*Arid4b*$^{PC-/-}$ mice at 5 months of age. Three mice from each genotype were analyzed. Data are means ± SEM. ***, $P < 0.001$. **c,** The mRNA levels of *Arid4b*, *p53*, and *Fas* of anterior prostates from the *Pten*$^{PC-/-}$ and *Pten*$^{PC-/-}$*Arid4b*$^{PC-/-}$ mice at 7 weeks of age were analyzed by qRT-PCR analyses. Three mice from each genotype were analyzed. The level of gene expression from one sample of the *Pten*$^{PC-/-}$ prostate was set as 1. Data are means ± SEM. *, $P < 0.05$; **, $P < 0.01$ ***, $P < 0.001$; Statistical analysis: *t*-test.

Supplementary Table 1. Features of prostate cell lines.

Cell line	Tumorigenicity	PTEN	Androgen sensitivity
PC3	High	-	Insensitive
DU145	Moderate	+	Insensitive
LNCaP	Low	-	Sensitive
C4-2B	High	-	Insensitive
RWPE-1	No	+	Sensitive
PZ-HPV-7	No	+	Sensitive
HPrEC	No	+	Sensitive

Supplementary Table 2. RNA-Seq analysis shows decreased expression of the AKT signaling downstream genes.

Gene	log_2FC SiARID4B/SiControl	P-value	Pathway
ADD3	-0.84	<0.001	mTORC1
FAM129A	-0.65	0.004	mTORC1
IGFBP5	-1.04	0.03	mTORC1
PSAT1	-1.39	<0.001	mTORC1
CCND1	-0.99	<0.001	FOXO and GSK3
KDM6B	-1.09	<0.001	TNFA signaling via NF-kB
FOSB	-1.38	0.02	TNFA signaling via NF-kB

Statistical analysis: Wald Chi-Squared test.

Supplementary Table 3. The tendency of mutual exclusivity between *ARID4B* and *PTEN* deletions. Analysis of five prostate cancer genome datasets using the cBioportal data source (http://www.cbioportal.org/) suggests a tendency of mutual exclusivity between *ARID4B* and *PTEN* deletions in prostate cancer genome. Statistical analysis: Fisher's exact test.

Cohort	Sample size	*ARID4B* alteration rate (n)	*PTEN* alteration rate (n)	Tendency	P value
Armenia, *et al.* *Nat Genetics* 2018	1,013	3% (30)	16 % (162)	Mutual exclusivity	0.500
TCGA, *Cell* 2015	333	4 % (13)	17 % (56)	Mutual exclusivity	0.599
Robinson, *et al.* *Cell* 2015	150	2.7 % (4)	40 % (60)	Mutual exclusivity	0.649
Kumar, *et al.* *Nat Med* 2016	54	9 % (4)	44 % (23)	Mutual exclusivity	0.110
Taylor, *et al.* *Cancer Cell* 2010	103	1 % (1)	14 % (14)	Mutual exclusivity	0.854

Statistical analysis: Fisher's exact test.

Supplementary Table 4. Validation of the *ARID4B/PIK3CA/PTEN* three-gene signature for prostate cancer recurrence using the GSE40272 dataset (n = 89).

Single gene validation for recurrence-free survival

Gene	Hazard ratio	P value
ARID4B	2.34	0.059
PIK3CA	0.90	0.790
PTEN	1.44	0.379

Two genes-combined validation for recurrence-free survival

Gene 1	Gene 2	Hazard ratio	P value
ARID4B +	*PIK3CA*	2.41	0.050
ARID4B +	*PTEN*	4.11	0.010
PIK3CA +	*PTEN*	1.16	0.720

Three gene-combined validation for recurrence-free survival

Gene 1	Gene 2	Gene 3	Hazard ratio	P value
ARID4B +	*PIK3CA* +	*PTEN*	4.58	0.006

The prognostic index (PI), also known as the risk score, is used to generate risk groups with classical multivariate Cox model. The Cox fitting is performed in R using the *survival* package. Higher PI represents higher risk, and the two risk groups (high and low) were generated by splitting patients with the median PI. When the patient PI was estimated with single gene (*ARID4B*, *PIK3CA* or *PTEN*) and corresponding explanatory variables, non-significant correlation with patient prognosis was found (*ARID4B*: HR: 2.34, P = 0.059; *PIK3CA*: HR: 0.9, P = 0.79; *PTEN*: HR: 1.44, P = 0.379). On the other hand, combination of *ARID4B* with *PIK3CA* or *PTEN* (combination of two genes) increases the hazard ratio with significant difference between high- and low-risk groups (*ARID4B* + *PIK3CA*: HR: 2.41, P = 0.05; *ARID4B* + *PTEN*: HR: 4.11, P = 0.01). Furthermore, the *PTEN*/*ARID4B*/*PIK3CA* three-gene combinatorial signature presents the most predictive power for tumor recurrence (HR: 4.58, P = 0.006) over that of individual gene alone or any two genes combined. Statistical analysis: log-rank test.

Supplementary Table 5. Multivariate Cox regression analysis on the correlations of *PTEN*, *ARID4B*, and *PIK3CA* with time to recurrence in prostate cancer patients in the GSE21032 dataset (n = 140).

Multivariate Cox	P value	HR	95% CI
ARID4B	0.677	1.15	0.6 - 2.21
PIK3CA	0.079	1.83	0.93 - 3.58
PTEN	0.012	2.45	1.22 - 4.93
ARID4B + PIK3CA	0.366	1.35	0.7 - 2.62
PTEN + ARID4B	0.015	2.38	1.19 - 4.76
PTEN + PIK3CA	0.013	2.42	1.21 - 4.86
PTEN + ARID4B + PIK3CA	0.002	3.23	1.56 - 6.72

Statistical analysis: log-rank test.

Supplementary Table 6. Multivariate Cox regression analysis on the correlations of *PTEN*, *ARID4B*, and *PIK3CA* with time to recurrence in prostate cancer patients in the TCGA provisional dataset (n = 492).

Multivariate Cox	P value	HR	95% CI
ARID4B	0.303	1.24	0.82 - 1.87
PIK3CA	0.793	1.06	0.70 – 1.59
PTEN	0.267	0.79	0.53 – 1.20
ARID4B + PIK3CA	0.257	1.27	0.84 – 1.91
PTEN + ARID4B	0.302	1.24	0.82 – 0.88
PTEN + PIK3CA	0.250	1.27	0.84 – 1.93
PTEN + ARID4B + PIK3CA	0.025	1.51	0.94 – 2.28

Statistical analysis: log-rank test.
