# Supplementary material for: Identification of the PTEN-ARID4B-PI3K pathway reveals the dependency on ARID4B by PTEN-deficient prostate cancer
Source: Nat Commun. 2019 Sep 24;10:4332. doi: 10.1038/s41467-019-12184-8 (PMC6760172; doi:10.1038/s41467-019-12184-8)
Supplement: Supplementary file 3 — Description of Additional Supplementary Files [file 41467_2019_12184_MOESM3_ESM.pdf]

Description of Additional Supplementary Files

File Name: Supplementary Data 1
Description: Upregulation or downregulation (P-value <0.05, |Log2FC| ≥ 0.59) of 1,419 and 1,757 genes, respectively, in PC3 cells with knockdown of ARID4B identified by RNA-Seq analysis. Statistical analysis: Wald Chi-Squared test.

File Name: Supplementary Data 2
Description: ChIP-Seq analysis with the anti-ARID4B antibody using PC3 cells identified 11,678 genes containing the ARID4B binding sites within 10 Kb of their gene margins.

File Name: Supplementary Data 3
Description: ARID4B directly regulated targets (1,555 genes) identified by both RNA-Seq (P-value <0.05, |Log2FC| ≥ 0.59) and ChIP-Seq analyses using PC3 cells.
