# Supplementary material for: Identification of the PTEN-ARID4B-PI3K pathway reveals the dependency on ARID4B by PTEN-deficient prostate cancer
Source: Nat Commun. 2019 Sep 24;10:4332. doi: 10.1038/s41467-019-12184-8 (PMC6760172; doi:10.1038/s41467-019-12184-8)
Supplement: Supplementary file 7 — Reporting Summary [file 41467_2019_12184_MOESM7_ESM.pdf]

nature research

Corresponding author(s): Ray-Chang Wu and Mei-Yi Wu

Last updated by author(s): Aug 9, 2019

Reporting Summary

Nature Research wishes to improve the reproducibility of the work that we publish. This form provides structure for consistency and transparency in reporting. For further information on Nature Research policies, see Authors & Referees and the Editorial Policy Checklist.

Statistics

For all statistical analyses, confirm that the following items are present in the figure legend, table legend, main text, or Methods section.

n/a	Confirmed	
☐	☒	The exact sample size (*n*) for each experimental group/condition, given as a discrete number and unit of measurement
☐	☒	A statement on whether measurements were taken from distinct samples or whether the same sample was measured repeatedly
☐	☒	The statistical test(s) used AND whether they are one- or two-sided<br>*Only common tests should be described solely by name; describe more complex techniques in the Methods section.*
☐	☒	A description of all covariates tested
☐	☒	A description of any assumptions or corrections, such as tests of normality and adjustment for multiple comparisons
☐	☒	A full description of the statistical parameters including central tendency (e.g. means) or other basic estimates (e.g. regression coefficient) AND variation (e.g. standard deviation) or associated estimates of uncertainty (e.g. confidence intervals)
☐	☒	For null hypothesis testing, the test statistic (e.g. *F*, *t*, *r*) with confidence intervals, effect sizes, degrees of freedom and *P* value noted<br>*Give P values as exact values whenever suitable.*
☒	☐	For Bayesian analysis, information on the choice of priors and Markov chain Monte Carlo settings
☒	☐	For hierarchical and complex designs, identification of the appropriate level for tests and full reporting of outcomes
☐	☒	Estimates of effect sizes (e.g. Cohen's *d*, Pearson's *r*), indicating how they were calculated

Our web collection on statistics for biologists contains articles on many of the points above.

Software and code

Policy information about availability of computer code

Data collection	Genomic alterations of ARID4B and PTEN from human prostate cancer datasets were acquired from The cBioPortal for Cancer Genomics (http://www.cbioportal.org). The mRNA expression z-Scores of ARID4B and PIK3CA with the corresponding Pearson correlation coefficient in prostate cancer samples in the TCGA database and the heat map with a 3-color scale set at number of −3, 0, and 3 were also obtained from The cBioPortal for Cancer Genomics (http://www.cbioportal.org).<br>Kaplan–Meier survival analysis was conducted using PROGgeneV2 prognostics database34 to identify that expression of ARID4B in prostate tumors is negatively associated with recurrence-free survival in prostate cancer patients from the GSE40272 and the Glinsky datasets. The multivariate Cox proportional hazards model of the ARID4B/PTEN/PIK3CA signature combines these three-gene expression levels with other clinical and pathological variables into an integrated risk score model to determine the correlations of ARID4B, PTEN, and PIK3CA with time to recurrence in prostate cancer patients from the two datasets (GSE21032and GSE40272) using SurvExpress web tool. According to this three-gene signature of ARID4B/PTEN/PIK3CA, Kaplan–Meier survival analysis was used to dichotomize prostate cancer cases with low and high risk for recurrence from the two datasets (GSE21032 and GSE40272).
Data analysis	BM SPSS Statistics Version 22 was used to analysis the data.

For manuscripts utilizing custom algorithms or software that are central to the research but not yet described in published literature, software must be made available to editors/reviewers. We strongly encourage code deposition in a community repository (e.g. GitHub). See the Nature Research guidelines for submitting code & software for further information.

Data

Policy information about availability of data

All manuscripts must include a data availability statement. This statement should provide the following information, where applicable:

- Accession codes, unique identifiers, or web links for publicly available datasets
- A list of figures that have associated raw data
- A description of any restrictions on data availability

RNA-Seq and ChIP-Seq raw data that support the findings of this study have been deposited to the Gene Expression Omnibus (https://www.ncbi.nlm.nih.gov/geo/) with the accession code GSE116670. All other data in this study are available from the corresponding author upon reasonable request.

Field-specific reporting

Please select the one below that is the best fit for your research. If you are not sure, read the appropriate sections before making your selection.

☒ Life sciences ☐ Behavioural & social sciences ☐ Ecological, evolutionary & environmental sciences

For a reference copy of the document with all sections, see nature.com/documents/nr-reporting-summary-flat.pdf

Life sciences study design

All studies must disclose on these points even when the disclosure is negative.

Sample size	Sample size were determined by taking into consideration previous experience with animal strains, assay sensitivity, tissue collection methodology used, and calculation of statistical significance between groups.
Data exclusions	No data were excluded from analyses.
Replication	All mouse experiments were performed using 3 – 16 mice. All experiments from cell lines were performed at least three independent experiments in triplicate. All these attempts at replication were successful.
Randomization	This is not relevant to my study. In all experiments, we used mutant mice and cells with their counterparts for the controls.
Blinding	This is not relevant to my study. In all experiments, we used mutant mice and cells with their counterparts for the controls.

Reporting for specific materials, systems and methods

We require information from authors about some types of materials, experimental systems and methods used in many studies. Here, indicate whether each material, system or method listed is relevant to your study. If you are not sure if a list item applies to your research, read the appropriate section before selecting a response.

Materials & experimental systems

n/a	Involved in the study
☐	☒ Antibodies
☐	☒ Eukaryotic cell lines
☒	☐ Palaeontology
☐	☒ Animals and other organisms
☒	☐ Human research participants
☒	☐ Clinical data

Methods

n/a	Involved in the study
☐	☒ ChIP-seq
☒	☐ Flow cytometry
☒	☐ MRI-based neuroimaging

Antibodies

Antibodies used	Anti-ARID4B (A302-233A, Bethyl Laboratories), anti-PIK3CA (C73F8; 4249, Cell Signaling Technology), anti-PIK3R2 (T15; sc-56934, Santa Cruz Biotechnology), anti-phospho-AKT (Thr308) (9275, Cell Signaling Technology), anti-phospho-AKT (Ser473) (9271, Cell Signaling Technology), anti-AKT (9272, Cell Signaling Technology), anti-phospho-mTOR (Ser2448) (2971, Cell Signaling Technology), anti-mTOR (7C10; 2983, Cell Signaling Technology), anti-CCND1 (M-20; sc-718, Santa Cruz Biotechnology), anti-HSP70 (3A3; sc-32239, Santa Cruz Biotechnology), anti-actin (AC-74; A2228; Sigma), anti-histone H1 (AE-4, sc-8030, Santa Cruz Biotechnology), anti-H3K4me3 antibody (07-473, EMD Millipore), anti-H4AC antibody (06-866, Upstate), anti-H3K27Ac antibody (ab4729, Abcam), anti-HDAC1 (05-614, Upstate), anti-PTEN (A2B1; sc-7974, Santa Cruz Biotechnology), and anti-Ki67 (B56; 550609, BD Biosciences).
Validation	Bethyl Laboratories<br>Anti-ARID4B: In addition to validation by the manufacturer, this antibody has been independently validated in the authors' labs by WB following using siRNA knockdown in cell lines and by immunostaining of mouse tissues from wild-type and Arid4b

knockout mice. In addition, for the manufacturer's validation statement please go to: https://www.bethyl.com/content/product-validation. This antibody has been cited in 4 publications.

Cell Signal Technology
Anti-PIK3CA (cited in 139 publications), anti-phosphoAKT(Thr308, cited in 642 publications), anti-phospho-AKT (Ser473, cited in 3073 publications), anti-AKT (cited in 3737 publications), anti-phosphomTOR (cited in 575 publications), anti-mTOR (cited in 656 publications): All these antibodies have been validated to detect the target proteins from mouse and human by the manufacturer in WB. For the validation statement please go to: https://www.cellsignal.com/contents/our-approach/cst-antibody-validation-principles/ourapproach-validation-principles.

Santa Cruz Biotechnologies: Polyclonal antibodies were validated using ELISA (anti-CCND1 and anti-AR) and monoclonal antibodies were validated by western blot (anti-PIK3R2, anti-HSP70, and anti-histone H1) by Santa Cruz Biotechnologies.
Anti-CCND1: cited in 260 publications, recognized target protein from human and mouse in WB.
Anti-HSP70: cited in 61 publications, validated by immunoprecipitation followed by WB, recognized target protein from human and mouse in WB.
Anti-PIK3R2: cited in 3 publications, recognized target protein from human and mouse in WB.
Anti-histone H1: cited in 245 publications used in WB, IF, and IHC(P).
anti-PTEN: cited in 305 publications used in WB, IF, IP, ELISA, and IHC(P).

Sigma
Anti-actin antibody: This antibody recognizes [illegible]-actin from many species, including human and mouse. This antibody has been cited in 1,056 publications.

EMD Millipore
Anti-H3K4me3: cited in 604 publications, validated by WB, ChIP, and ChIP-seq.
Anti-H4Ac: cited in 837 publications, validated by WB, ChIP, IP, and IHC.

Abcam
Anti-H3K27Ac: cited in 769 publications, validated by WB, ChIP, ICC-IF, and IHC.

BD Biosciences
Anti-Ki67: cited in 187 publications, recognized target protein from human and mouse in IHC.

Eukaryotic cell lines

Policy information about cell lines

Cell line source(s)	PC3 (ATCC® CRL-1435™), DU 145 (ATCC® HTB-81™), LNCaP (ATCC® CRL-1740™), C4-2B (ATCC® CRL-3315™), RWPE-1 (ATCC® CRL-11609™), PZ-HPV-7 (ATCC® CRL-2221™), HPrEC(ATCC® PCS-440-010™), and HeLa (ATCC® CCL-2™) are purchased from the American Type Culture Collection (ATCC).
Authentication	All cell lines were authenticated by ATCC.
Mycoplasma contamination	All cells were tested negative for mycoplasma contamination.
Commonly misidentified lines (See ICLAC register)	No commonly misidentified cell lines were used in this study.

Animals and other organisms

Policy information about studies involving animals; ARRIVE guidelines recommended for reporting animal research

Laboratory animals	Mice with prostate-specific deletion of Pten (PtenPC-/-) contain the Pb-Cre (the Cre gene under control of the prostate-specific probasin promoter) and conditional knockout Ptenflox/flox alleles. The Ptenflox/flox mice and Pb-Cre mice were backcrossed to generate the PtenPC-/- mice. The PtenPC-/- mice were further backcrossed with the Arid4bflox/flox mice to generate the PtenPC-/-Arid4bPC-/- mice that carry prostate-specific deletions of Pten and Arid4b containing the Pb-Cre27, Ptenflox/flox, and Arid4bflox/flox alleles. Male mice were used at 7 weeks to 5 months of age.
Wild animals	This study did not involved wild animals.
Field-collected samples	Experimental mice and studies were approved by the Institutional Animal Care and User Committee (IACUC) of The George Washington University (protocol number: A315). All of the mice were bred and maintained at the institution's specific pathogen-free mouse facility. The facility is approved by the American Association for Accreditation of Laboratory Animal Care and operated in accordance with current regulations and standards of the US Department of Agriculture and the Department of Health and Human Services.
Ethics oversight	Experimental animals and studies were approved by the Institutional Animal Care and User Committee (IACUC) of The George Washington University (protocol number: A315). All of the mice were bred and maintained at the institution's specific pathogen-free mouse facility. The facility is approved by the American Association for Accreditation of Laboratory Animal Care and operated in accordance with current regulations and standards of the US Department of Agriculture and the Department of Health and Human Services.

Note that full information on the approval of the study protocol must also be provided in the manuscript.

ChIP-seq

Data deposition

☒ Confirm that both raw and final processed data have been deposited in a public database such as GEO.

☒ Confirm that you have deposited or provided access to graph files (e.g. BED files) for the called peaks.

Data access links<br>*May remain private before publication.*	ChIP-Seq raw data have been deposited to the Gene Expression Omnibus (https://www.ncbi.nlm.nih.gov/geo/) with the accession code GSE116670. To review GEO accession GSE116670: Go to https://www.ncbi.nlm.nih.gov/geo/query/acc.cgi?acc=GSE116670. Enter token szmfsyqmtjmjlcl into the box.
Files in database submission	RAW FILES:<br>1_005JGWU_PC3_ARID4B_hs_i79.fastq<br>2_005JGWU_PC3_Input_hs_i75.fastq<br><br>PROCESSED DATA FILES:<br>005JGWU_ARID4B_PC3_ucsctracks.bed<br>005JGWU_ARID4B_PC3_bigWig_URLs.txt
Genome browser session<br>(e.g. UCSC)	UCSC: https://genome.ucsc.edu/

Methodology

Replicates	The ARID4B ChIP data were acquired from single experiment of PC3 cells, and the replicate agreement is not applicable.
Sequencing depth	The total number of reads: 29,025,160 (input) / 34,839,290 (ARID4B ChIP);<br>Total number of alignments (hg19): 25,822,346 (input) / 32,687,482 (ARID4B ChIP);<br>Unique alignments (-q 25): 23,294,855 (input) / 29,680,626 (ARID4B ChIP);<br>Unique alignments (without duplicate reads): 22,074,127 (input) / 22,008,215 (ARID4B ChIP);<br>Final number of tags (-chrM, >chromsize): 22,053,529 (input) / 21,995,197 (ARID4B ChIP);<br>Normalized tags: 21,995,197 (input) / 21,995,197 (ARID4B ChIP);<br>Read length: single-end 75 bp
Antibodies	Anti-ARID4B (A302-233A, Bethyl Laboratories).
Peak calling parameters	The two main peak callers used at Active Motif, inc. who we appointed to conduct and file the sequencing, and the peak calling process are MACS 1.4.2 and SICER. The parameters are listed as follows:<br><br>Program Settings<br>NCBI Build for Affymetrix Arrays : 37_2<br>NCBI Build for Analysis after conversion: 37_2<br>Sample Name #1: 1_PC3_ARID4B::1<br>Intervals File for 1_PC3_ARID4B::1: MACS/1_PC3_ARID4B_hg19_peaks_filtered.bed<br>BAR File for Sample 1_PC3_ARID4B::1: SEQ/1_005JGWU_PC3_ARID4B_hg19_i79_uniqnorm_signal.txt<br>Promoter Start: -7,500<br>Promoter End: 2,500<br>Intragenic Region Start: 1,000<br>Max Distance between Intervals in an Active Region: 0<br>Gene Margin Upstream of gene: 10,000<br>Gene Margin Downstream of gene: 10,000<br>CpG Island Margin: 200<br>Promoter Start Margin: -7,500<br>Promoter End Margin: 2,500<br>Gene Tx Start Margin for NCBI Genes 500<br><br>Input Parameters<br>1_PC3_ARID4B::1::BarFile SEQ/1_005JGWU_PC3_ARID4B_hg19_i79_uniqnorm_signal.txt<br>1_PC3_ARID4B::1::IntFile MACS/1_PC3_ARID4B_hg19_peaks_filtered.bed<br>AffyBuild 37.2<br>AllowedContigReference GRCh37.p2-Primary Assembly<br>AnnotateList CpG<br>BaseDir /Users/paullabhart/Genpathway/Systems/CSeq/GWU/005J<br>BlatDB hg19<br>Build 37.2<br>ClusterMargin 0<br>CombinedWorkbook 2<br>CpGMargin 200<br>CSeq 1<br>CSeqBGWindowSize 20000<br>CSeqBinSize 32<br>CSeqFiles SEQ/1_005JGWU_PC3_ARID4B_hg19_i79_uniqnorm, SEQ/2_005JGWU_PC3_Input_hg19_i75_uniqnorm

CSeqFragSize 200
CSeqMachine bed
CSeqMaxGap 100
CSeqMinRun 2
Experiment CSeq 005J GWU
FactorPath 1
GeneMarginDown 10000
GeneMarginUp 10000
GeneStartMargin 500
MACS2Broad 0
MACS2BroadCutoff 0.1
MACS2MFold 5 50
MACSBandwidth 200
MACSControlFiles SEQ/2_005JGWU_PC3_Input_hg19_i75_uniqnorm_hits.bed
MACSGenomeSize hs
MACSMFold 10,30
MACSName 1_PC3_ARID4B_hg19
MACSNoLambda 0
MACSNoModel 0
MACSPValue 0.0000001
MACSTagSize 75
MACSTreatmentFiles SEQ/1_005JGWU_PC3_ARID4B_hg19_i79_uniqnorm_hits.bed
NoTranslation 1
OutFileBase Analysis/005JGWU_ARID4B_PC3
OutTableList intervals, genes, clusters
PeakCallTool MACS
PeakHoodLength 60
PeakHoodMaxNum 1000
PeakSamples 1_PC3_ARID4B::1
PrintList intervals, genes, chromosomes, stats, clusters
ProbeLength 1
PromoterEnd 2500
PromoterStart -7500
RNAStartMargin 500
Samples 1_PC3_ARID4B::1
StandardChromosomes T
TaxID hs

MACS1.4.2
effective genome size = 2.70e+09
band width = 200
model fold = 10,30
# pvalue cutoff = 1.00e-07

Data quality

In sequencing analysis, only reads that pass Illumina's purity filter, align with no more than 2 mismatches, and map uniquely to the genome are used in the subsequent analysis. In addition, duplicate reads ("PCR duplicates") are removed. All reads from FASTQ files were Illumina chastity-filtered with phred+33 quality scores.
The Final MACS peaks number is 10,066, including 119 negative peaks (Empirical FDR = 1.18%) and 3 ENCODE blacklisted peaks.

Software

MACS 1.4.2 & SICER
